# Preventing healthcare-associated infection in Switzerland: Results of a national survey

**DOI:** 10.1017/ice.2019.351

**Published:** 2020-05

**Authors:** Hugo Sax, Peter W. Schreiber, Lauren Clack, David Ratz, Sanjay Saint, M. Todd Greene, Stefan P. Kuster

**Affiliations:** 1 Department of Infectious Diseases and Hospital Epidemiology, University Hospital Zurich, University of Zurich, Zurich, Switzerland; 2 Veterans’ Affairs Center for Clinical Management Research, VA Ann Arbor Healthcare System, Ann Arbor, Michigan, United States; 3 Department of Internal Medicine, University of Michigan Medical School, Ann Arbor, Michigan, United States; 4 Veterans’ Affairs/University of Michigan Medical School Patient Safety Enhancement Program, Ann Arbor, Michigan, United States

## Abstract

We assessed infection prevention in Swiss hospitals via a national survey focusing on infection prevention practices prior to a large national infection prevention initiative. Of the 59 hospitals that responded (77%), 98% had infection prevention teams and 40% very good or excellent leadership support. However, a minority of hospitals used recommended infection prevention practices and surveillance systems regularly.

Healthcare-associated infection (HAI) is a major concern worldwide. In Europe an estimated 3.4 million patients are affected by HAI annually.^1^ The Federal Offices of Public Health and the Swiss Center for Infection Prevention Swissnoso are currently leading a national program, “NOSO Strategy” (https://www.bag.admin.ch/bag/en/home/strategie-und-politik/nationale-gesundheitsstrategien/strategie-noso--spital--und-pflegeheiminfektionen/ueber-die-strategie.html), to reduce infection risk in the Swiss healthcare system through governance, monitoring, prevention, education, and research.

Catheter-associated urinary tract infection (CAUTI), ventilator-associated pneumonia (VAP), central-line–associated bloodstream infection (CLABSI), and surgical site infection (SSI) are prime targets for most infection prevention programs because invasive procedures represent modifiable risk factors. Additionally, *Clostridioides difficile* infection (CDI) has caused repeated outbreaks in hospitals globally, and organizational culture is an important precondition for successful implementation of good infection prevention practice.^2^ We investigated the status of structural, procedural, and cultural aspects of infection prevention in Swiss midsize-to-large acute-care hospitals prior to the rollout of the NOSO Strategy.

## Methods

Between October 2015 and March 2017, all Swiss acute-care general and children’s hospitals with ≥3,000 annual discharges (https://www.bfs.admin.ch/bfs/de/home/statistiken/kataloge-datenbanken/publikationen.assetdetail.169879.html) were invited to participate. Nonresponders received phone and e-mail reminders.

Data were collected using a questionnaire developed by Saint et al^3^ that covers general hospital characteristics, infection prevention policies, infection prevention staffing, and use of specific practices related to surveillance and prevention of CAUTI, CLABSI, VAP, and CDI. The survey was translated to German, French, and Italian, and was pretested by infection prevention specialists for the final online version (Survey Monkey, San Mateo, CA).

Descriptive statistics, N (%) for categorical variables, and mean (±SD) or median (range) for continuous variables were calculated for hospital characteristics and specific infection prevention practices using SAS version 9.4 software (SAS Institute, Cary, NC) and Stata/SE version 14.2 software for Mac (StataCorp, College Station, TX). The 2 affirmative values of incremental rating scales were expressed as proportions of positive answers. “Don’t know” was recoded as negative. Missing answers were excluded.

According to the Swiss law on research on humans, ethics approval was waived. Responses remained confidential.

## Results

Overall, 77 hospitals met the inclusion criteria and were invited; 59 hospitals (77%) responded, mainly through infection prevention nurse practitioners (93%). Of these hospitals, 3 were university hospitals (return rate, 60%), 3 were children’s hospitals (100%), 26 (77%) were secondary care centers, and 27 (71%) were primary care centers; 10 (return rate, 77%) were French-speaking hospitals, 47 (77%) were German-speaking hospitals, and 2 (67%) were Italian-speaking hospitals.

### Infrastructure

Select hospital and infection prevention infrastructure characteristics are displayed in Supplementary Table 1 (online). All hospitals reported having infection prevention policies. All but 1 hospital had infection prevention professional(s). Infection prevention staffing varied markedly; only 16 (27%) hospitals met the requirement for 1 infection prevention nurse per 100 acute-care beds.^2^

### Culture

Although most respondents (48 of 58, 83%) felt that hospital leadership encouraged putting patient safety at the center of efforts, only 23 of 58 (40%) reported having very good or excellent support from hospital leadership for infection prevention (Supplementary Fig. 1 online).

### Practices

**Fig. 1.**
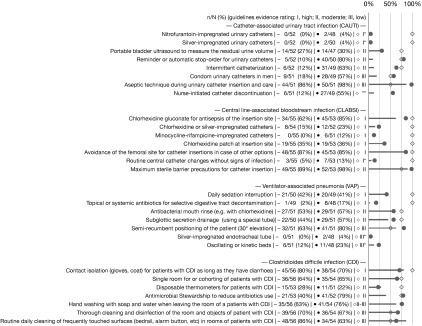
Use of prevention practices and perception of the strength of supporting evidence. The line graphs represent the use of preventive practices. Lines represent the level of practice; dots represent the perception of the supporting evidence; diamonds represent the evidence form authoritative infection prevention guidelines. A high level of evidence appears at 100%, moderate level at 75%, and a low level at 50% (for references, see the text). For prevention practices, 5-point scales from “never” to “always” were transformed into a dichotomous variable recoding “almost always” and “always” as 1 (“yes”) and the remainder as 0 (“no”). Missing answers and “don’t know” were excluded from the calculation of proportions. Note. *C. difficile*, *Clostridioides difficile*. *Not recommended practice. **No evidence level reported in guidelines.

Median use of recommended infection prevention practices was 18% (range, 10%–86%) for CAUTI, 48.5% (range, 0%–89%) for CLABSI, 44% (range, 2%–63%) for VAP, and 64% (range, 40%–86%) for CDI (Fig. 1). In general, perceived evidence matched practice use with the exception of CAUTI, for which perceived evidence strength mismatched published evidence strength. Indications for urinary catheters included prolonged surgery in 46 of 51 hospitals (90%), urinary output monitoring in 41 of 52 hospitals (79%), urinary obstruction in 33 of 52 hospitals (63%), skin ulceration in 8 of 52 hospitals (15%), incontinence in 7 of 51 hospitals (14%), nursing workload in 1 of 52 hospitals (2%), and patient or family wish in 0 of 50 hospitals (0%). Use of a central-line insertion checklist was reported by 37 of 54 hospitals (69%), an insertion kit was used in 46 of 55 hospitals (84%), and reassessment of central-line indication during clinical rounds occurred in 44 of 52 hospitals (85%). Early mobilization for VAP prevention was applied by 43 of 49 hospitals (88%).

### Surveillance

**Fig. 2.**
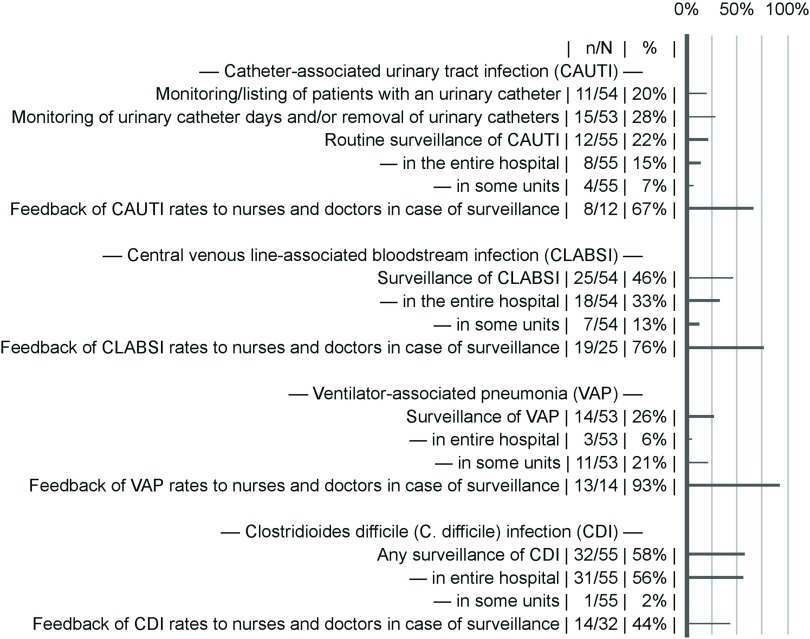
Healthcare-associated infection surveillance activities. Feedback of infection rates to healthcare providers was coded as present when it was indicated as at least given to some units of the hospital. Missing answers were excluded from the calculation of proportions.

Surveillance varied considerably by HAI type and hospital (Fig. 2). Local surveillance schemes for at least 1 HAI were reported by 34 of 59 hospitals (58%). Repeated HAI prevalence surveys were performed in 16 of 59 hospitals (27%). SSI surveillance was almost universal through the national surveillance scheme 57 of 59 hospitals (97%). Hospital-acquired pneumonia was surveilled in 7 of 59 hospitals (12%).

Applying multivariable models, we found no relevant correlations between staffing levels, leadership support, and perceived importance of HAI prevention with various infection prevention practices and monitoring activities (data not shown).

## Discussion

We conducted a comprehensive, national survey of Swiss acute-care hospitals to ascertain the state of infection prevention structure, practices, and organizational culture to prevent the most common HAIs prior to the rollout of a national infection prevention program.

Our study has several main findings. First, almost all Swiss general acute care hospitals had an infection prevention practitioner or team and infection prevention policies. However, the staffing of infection prevention teams in most hospitals did not meet the latest international recommendations.^4–7^ Second, perceived hospital leadership support for infection prevention was modest in most hospitals. Third, only a minority of hospitals sustained surveillance systems for HAI. Finally, specific prevention practices were implemented to varying degrees, being low for CAUTI and higher for CDI prevention.

Similar versions of the questionnaire^3^ distributed in the present survey have been distributed in other countries. A random sample of 900 US acute-care hospitals reported higher use of most prevention practices than Switzerland. In contrast to this study, the perceived importance of preventing specific HAIs and the certification status of the infection prevention leader were significantly associated with the use of prevention practices in US hospitals.^3^ Findings among Thai and Japanese hospitals suggested that increased organizational commitment toward safety culture and participating in infection prevention collaborative networks contribute to improvements in HAI prevention.^8^^,^^9^ In line with our findings, a European survey among 309 hospitals found that most hospitals featured CLABSI surveillance.^10^

Importantly, we identified a gap between perceived solid evidence to support many CAUTI prevention practices, especially system-level elements (eg, catheters reminders and nurse-initiated stop orders and use of alternative catheterization), consistent with low perceived awareness of CAUTI by hospital leadership. However, perceived strength of evidence for many practices mismatched the guideline-reported strength of evidence but matched actual use, a correlation found by others.^11^ This finding might just reflect the lacunar nature of research against common sense, demonstrated by aseptic technique during urinary and central-line insertion, for which the actual evidence is low^6^ to moderate^4^ although it was perceived as being high and was followed by a high level of use.

Whereas respondents perceived high levels of encouragement for patient safety, they felt less confident that their colleagues would be open to changes or that it would be easy to implement evidence-based guidelines. Perhaps most notably, only a minority felt that leadership support for infection prevention was very good to excellent. We also found that the level of perceived leadership interest did not always match the application of practices.

Our study has limitations. First, survey findings depend on the person responding. We sought to mitigate this by asking the respondent to crosscheck their opinions with knowledgeable colleagues. Second, surgical site infections and other infection prevention topics were not covered in this survey, as we kept the number of survey questions manageable in favor of a high return rate.

In conclusion, this comprehensive survey of infection prevention structure, practices, and organizational culture revealed a need for better infection prevention staffing in most Swiss acute-care hospitals and increased application of evidenced-based infection prevention practices and surveillance; this was especially so for CAUTI prevention. Although the presence of infection prevention teams and patient safety culture were rated relatively high, support for infection prevention by hospital leadership can likely be improved. The Swiss national infection prevention program will ideally address the specific hospital-level deficiencies noted in this national survey.

## Supplementary material

For supplementary material accompanying this paper visit http://dx.doi.org/10.1017/ice.2019.351. click here to view supplementary material
