# Supplementary material for: Preventing healthcare-associated infection in Switzerland: Results of a national survey
Source: Infect Control Hosp Epidemiol. 2020 Apr 13;41(5):597–600. doi: 10.1017/ice.2019.351 (PMC7199278; doi:10.1017/ice.2019.351)
Supplement: Supplementary file 1 [file S0899823X19003519sup001.docx]

Annex Table. Infection Prevention Infrastructure and Participation in National Programs in Swiss Acute Care Hospitals (N=59)

| Acute care beds; mean (standard deviation) | 253.8 (234.0) |
| --- | --- |
| Hospital has at least one part time dedicated infection prevention (IP) person or team; n/N (%) | 58/59 (98%) |
| Hospital has a hospital epidemiologist; n/N (%) | 45/59 (76%) |
| Hospital has a contract with a hospital epidemiologist; n/N (%) | 28/51 (55%) |
| Fulltime equivalent dedicated to IP; median (range) | 1.0 (0-10.0) |
| Hospital has IP policies; N (%) | 59/59 (100%) |
| Responsible for IP is formally trained in epidemiology and/or IP; N (%) | 49/58 (84%) |
| Participation in at least one regional/national/international IP programs/collaboratives; N (%) | 57/59 (97%) |
| Swissnoso Surgical Site Infection Surveillance Module; N (%) | 57/59 (97%) |
| German Krankenhaus Infektions Surveillance System; N (%) | 5/59 (9%) |
| Clean Hands Monitoring System (Handheld Device App with national database); N (%) | 27/59 (46%) |
| Progress Safe Surgery program; N (%) | 18/59 (31%) |

Annex Figure. Organizational culture regarding infection prevention and patient safety

[separate file]

Legend: n, number of respondents who responded positively; N, number of overall respondents for this item; %, proportion of n/N. For healthcare provider behavior, the 5-point incremental scale was dichotomously transformed by recoding “agree completely” and “agree” as 1 (“yes”) and the remainder as 0 (“no”). For leadership support, 4-point incremental scales were dichotomously transformed symmetrically. Missing and “unknown” answers were excluded from the analysis of proportions.
